# Chromosome-Level Genome Assembly and Genomic Analysis of the Hybrid Grouper ShanHu (*Epinephelus fuscoguttatus* ♀ × *Epinephelus polyphekadion* ♂)

**DOI:** 10.3390/ijms26115036

**Published:** 2025-05-23

**Authors:** Yiqun Liu, Yunxiang Mao

**Affiliations:** 1 Key Laboratory of Tropical Aquatic Germplasm of Hainan Province, Sanya Oceanographic Institute, Ocean University of China, Sanya 572000, China; liu1287414908@163.com; 2 Yazhou Bay Innovation Institute, Hainan Tropical Ocean University, Sanya 572022, China

**Keywords:** *Epinephelus fuscoguttatus*, *Epinephelus polyphekadion*, hybridization, chromosomal assembly, genome annotation, genome sequencing

## Abstract

Groupers are important aquaculture species, and hybridization is an effective breeding method for genetic improvement and to enhance production efficiency in groupers. The ShanHu grouper (*Epinephelus fuscoguttatus* ♀ × *Epinephelus polyphekadion* ♂) is a hybrid grouper with potential for aquaculture development and research value. Using Illumina and PacBio sequencing platforms, as well as PacBio SMRT technology and Hi-C auxiliary mounting technology, the whole genome sequencing and assembly of the ShanHu grouper were completed, resulting in a chromosome-level genome information for this hybrid grouper. The genome assembly has a total length of 1.17 Gb with a scaffold N50 of 46.12 Mb, and 171 contigs were anchored into 24 chromosomes. Additionally, its repeat sequences and non-coding RNAs were annotated and 26,102 genes were predicted. Through comparative genomic analysis of the hybrid species ShanHu grouper and its parents, we found that comparative genomic analyses revealed centric inversion structural variations on the chromosomes of the hybrid ShanHu grouper in relation to the brown-marbled grouper and the camouflage grouper. Furthermore, the gene families of the hybrid species have expanded in pathways related to immunity and growth development. This study is the first to provide complete genomic information for a hybrid grouper, offering its full genetic information, exploring the genetic variations in the genomes of hybrid offspring, and providing data references for foundational theoretical research and grouper production practices.

## 1. Introduction

Groupers (Epinephelidae, Perciformes) are important marine aquaculture fish consisting of 16 genera and approximately 160 species [1], among which approximately 47 species are currently farmed [2]. Currently, China’s annual marine aquaculture production of groupers has exceeded 200,000 tons [3]. Groupers also serve as good models for the study of marine ecological systems, social relations, and sex inversion [4].

Currently, the genomic research of groupers is progressing rapidly and the complete genome information of various economic groupers has been decoded. Examples are: *Epinephelus lanceolatus*, *E. akaara*, *Plectropomus leopardus*, *E. tukula*, *Cromileptes altivelis*, *E. cyanopodus*, *E. moara*, and *E. polyphekadion* [5,6,7,8,9,10,11,12]. The brown-marbled grouper (*E. fuscoguttatus*) and the camouflage grouper (*E. polyphekadion*) are two species with relatively mature aquaculture techniques. The brown-marbled grouper is known for its high disease resistance and stress tolerance [13]. Additionally, it has an extended spawning season of 8–9 months per year, with a stable spawning period and high-quality, high-volume egg production. Thus, it is considered an excellent maternal parent for hybridization with other grouper species, such as *E. fuscoguttatus* ♀ × *E. lanceolatus* ♂ and *E. fuscoguttatus* ♀ × potato grouper (*E. tukula*) ♂ [14,15]. The camouflage grouper, on the other hand, is characterized by its docile nature, high protein content, and delicious taste [16]. Hybridization is an effective breeding method used in fish farming to improve genetic traits and production efficiency. It is also one of the most widely used breeding technologies in fish breeding, producing offspring with traits superior to their parents through genetic recombination [17]. The ShanHu grouper (*E. fuscoguttatus* ♀ × *E. polyphekadion* ♂), created by using the brown-marbled grouper as the maternal parent and the camouflage grouper as the paternal parent, is an example of a well-developed hybrid grouper species with great commercial development potential and value for foundational theoretical research [18]. As a hybrid grouper, the ShanHu grouper integrates the advantageous traits of its parental generations. It demonstrates the characteristics of strong stress resistance and rapid growth rate from its paternal parent, as well as the features of high protein content and delicious flavor from its maternal parent. The ShanHu grouper represents enormous economic value for the industry. In the aspect of scientific research, the ShanHu is also an excellent model for studying the hybridization mechanism of groupers [19].

Whole genome sequencing represents a complete set of genetic information for an organism. A high-quality genome sequence is a crucial foundation and valuable resource for genomics, functional genomics, comparative genomics, species evolution, and gene function research [20]. Using third-generation PacBio SMRT sequencing technology and Hi-C high-throughput chromatin conformation we completed the chromosome-level whole genome assembly and annotation of the hybrid ShanHu grouper, acquiring a high-quality and complete set of genetic information for the hybrid species. On the whole-genome level we explored the differences between the brown-marbled grouper, the camouflage grouper, and their hybrid the ShanHu grouper. The whole genome sequencing results of the hybrid grouper provide a comprehensive genetic reference for future research on groupers.

## 2. Results

### 2.1. Genome Assembly and Quality Assessment

**Figure A1:**
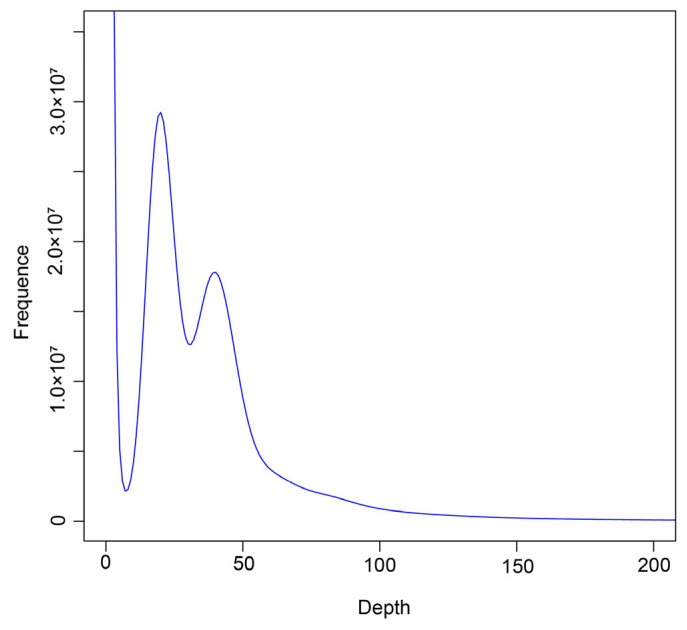
Distribution graph of *K*-mer for ShanHu. The horizontal axis represents the depth when *K*-mer = 17 and the vertical axis represents the frequency at different depths.

**Figure A2:**
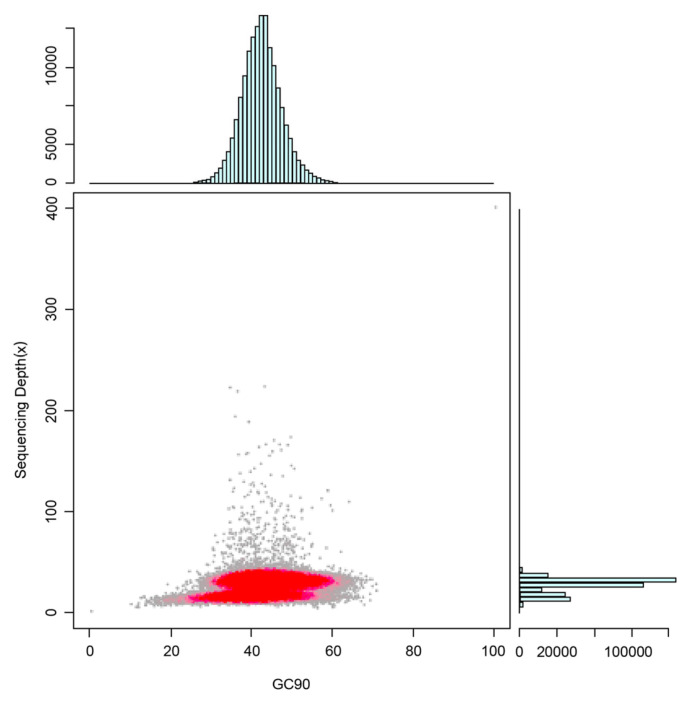
GC-depth distribution graph for ShanHu.

Using Illumina libraries and paired-end (PE) sequencing based on the Illumina Hiseq platform, we assessed the genome size, GC content, heterozygosity, repetitive sequences, and potential foreign contamination of the hybrid ShanHu groupers. First, a total of 59.30 Gb of raw data were obtained from the hybrid ShanHu groupers second-generation sequencing, with a sequence efficacy of 85.16%, an error rate of 0.04%, and Q20 and Q30 values of 96.72% and 91.52%, respectively. The GC content was found to be 41.38%. *K*-mer analysis was performed to evaluate the genome size and heterozygosity of the ShanHu groupers, setting the *K*-mer length to 17 bp (Figure A1). The predicted genome size of the hybrid ShanHu groupers was 1125.98 Mbp, with a heterozygosity of 1.7% and a repeat rate of 41.12%. Additionally, after assembling the data of the ShanHu groupers, the correlation results of the GC content and sequencing depth for the contigs are shown in Figure A2. The GC content of the contigs did not exhibit separation, indicating that the ShanHu groupers genome is free from foreign contamination.

The sequencing amount for ShanHu groupers based on PacBio technology was 100 Gb. Based on the estimated genome size of 1125 Mb from the second-generation sequencing data, the sequencing coverage depth was calculated to be 88.89×. After quality control of the sequencing data, we obtained the genome sequence of ShanHu groupers and conducted a preliminary assembly of its genome. The assembled genome sequence contained 153 contigs, with a contig N50 of 46.12 Mb and a total genome size of 1.17 Gb where the longest contig was 70.4 Mb.

**Figure A3:**
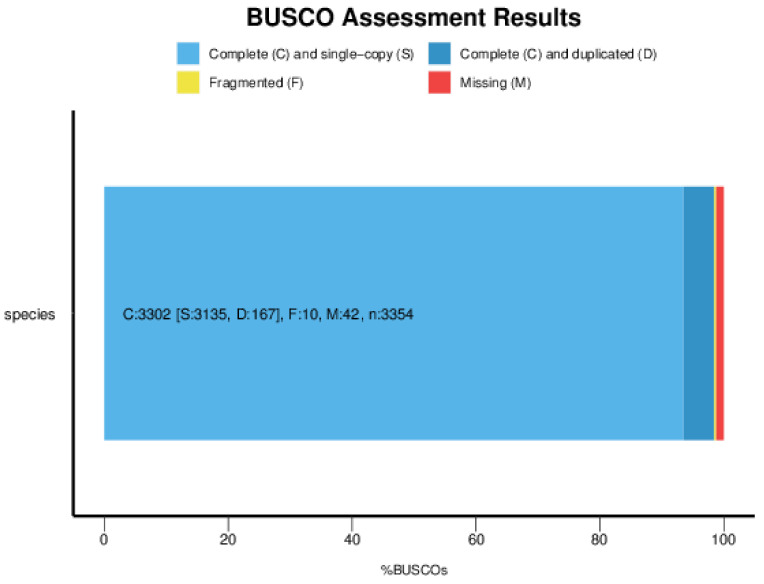
BUSCO assessment results for ShanHu.

Once the genome assembly of ShanHu groupers was completed, we assessed the quality of the assembly using BUSCO analysis to evaluate the genome integrity. The BUSCO evaluation results indicated that, using a single-copy ortholog gene set of 3354 genes, 98.5% of complete BUSCO genes were assembled, with 3135 complete single-copy genes successfully assembled from the ortholog gene set (Figure A3).

### 2.2. Pseudochromosome Construction

**Figure 1:**
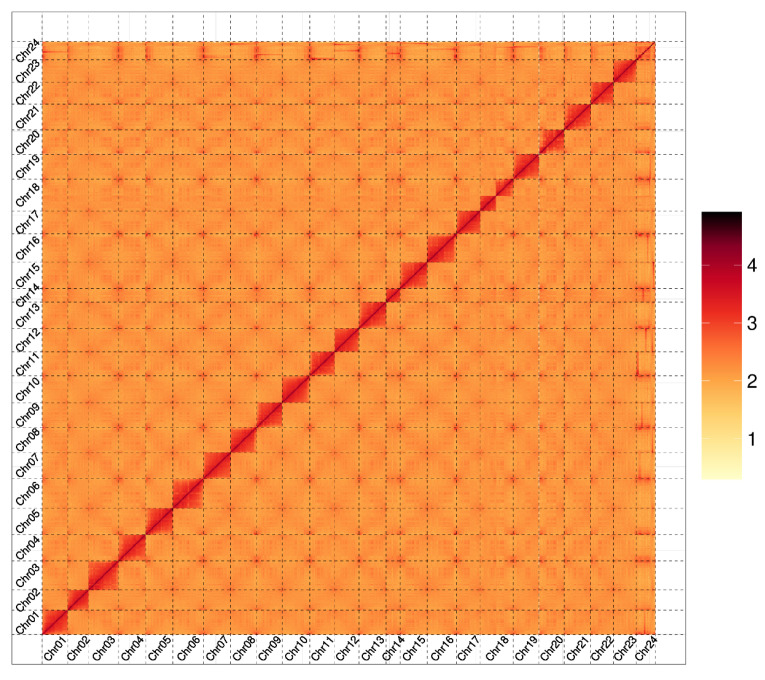
Hi-C contact map of the ShanHu genome. The color bar indicates contact density from yellow (low) to red (high).

HI-C assisted assembly was performed on the ShanHu groupers genome to achieve chromosome-level resolution (Figure 1). The chromosome-level genome assembly results are as follows (Table 1): the total length of contigs is 1,176,882,344 bp, with a contig N50 length of 45,365,500 bp; the total length of scaffolds is 1,176,892,644 bp, with a scaffold N50 length of 46,120,958 bp; and the total length of sequences anchored to the chromosomes is 1,083,187,597 bp, with 92.04% of the sequences anchored to the 24 chromosomes of the ShanHu groupers (Table A1).

### 2.3. Genome Annotation

**Figure 2:**
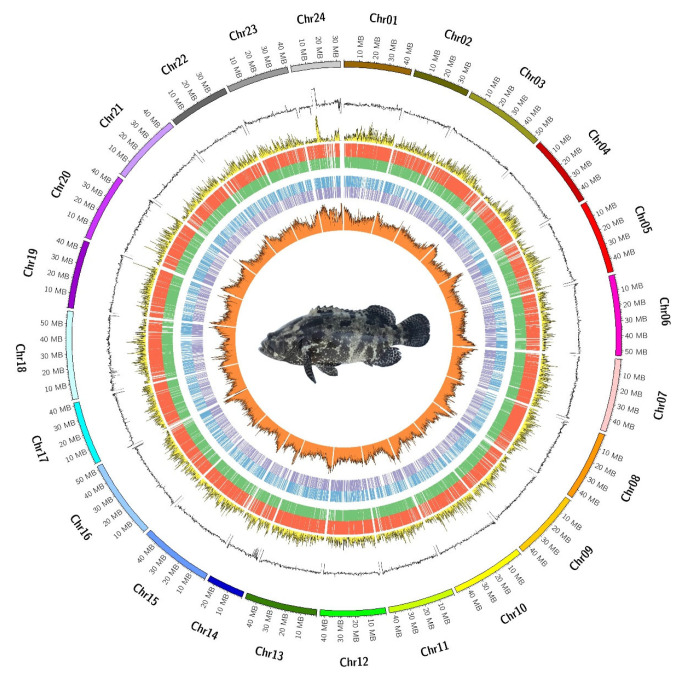
Genetic information characteristic diagram of the ShanHu genome. From the outer circle to the inner circle: 1 represents chromosomes of the ShanHu genome; 2 GC content; 3 gene density (yellow); 4 genes on the forward strand (red); 5 genes on the reverse strand (green); 6 non-coding RNA on the forward strand (blue); 7 non-coding RNA on the reverse strand (purple); 8 repeat sequence content (orange).

**Figure A4:**
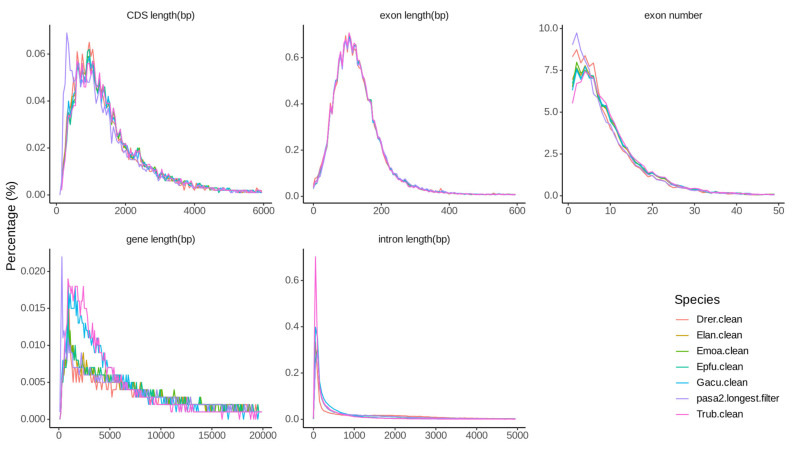
Comparison of gene structure components between ShanHu and annotated species.

**Figure A5:**
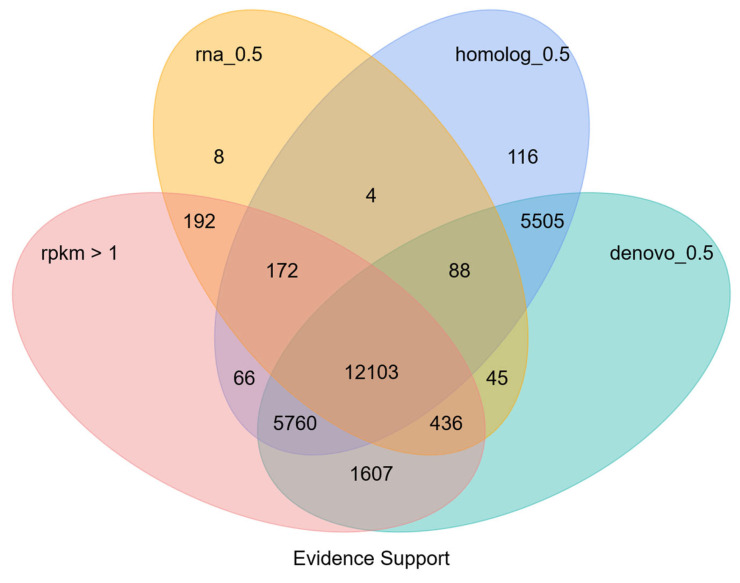
Venn diagram of gene structure annotation for ShanHu based on de novo prediction methods, homology prediction methods, and transcriptome data.

**Figure A6:**
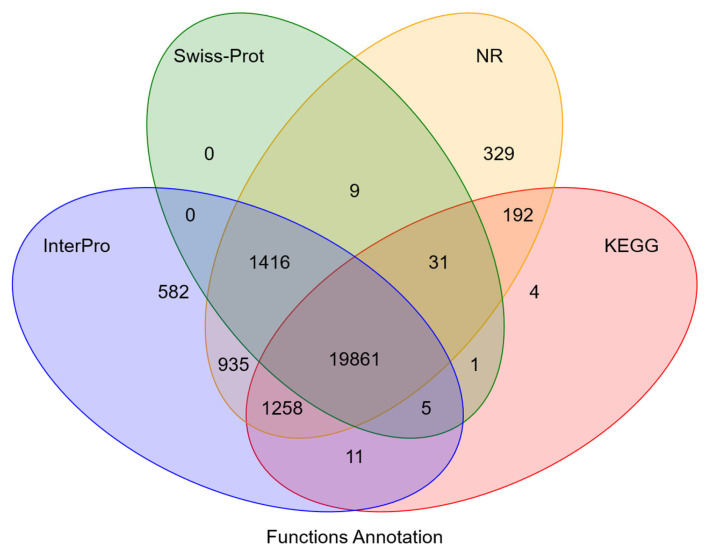
Venn diagram of gene function annotation statistics for ShanHu based on known protein databases.

Based on the assembled chromosome-level genome of the hybrid ShanHu groupers, we completed the annotation of repetitive sequences, non-coding RNAs, and protein-coding genes, as well as predicted related functions (Figure 2). For protein-coding genes, the ShanHu groupers genome predicted a total of 26,102 genes (Table 2) (Figure A4 and Figure A5), and based on comparisons with known protein databases 98.5% of the genes were able to have their functions predicted (Figure A6). Repeat sequences were identified using homologous and de novo methods, revealing a total of 552.11 Mbp of repetitive sequences in the ShanHu groupers genome, which accounts for 46.91% of the genome (Table 3). For the non-coding RNAs in the ShanHu groupers, a total of 3438 miRNAs, 2415 tRNAs, 8374 rRNAs, and 1671 snRNAs were identified (Table 4).

### 2.4. Phylogenetic Analysis of Economically Epinephelus

**Figure 3:**
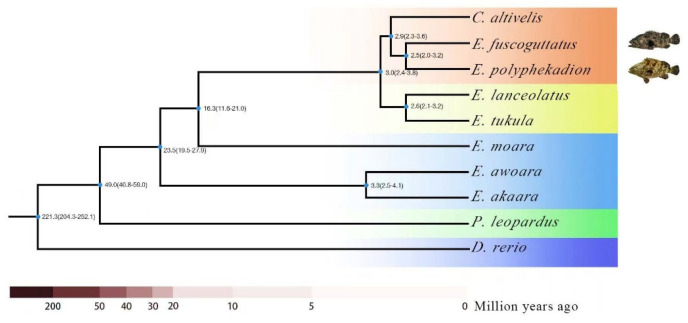
Phylogenetic tree of economically important Epinephelus. The numbers at the nodes represent the divergence time of the grouper ancestors in millions of years, with the numbers in parentheses indicating the confidence range of that divergence time.

**Figure A7:**
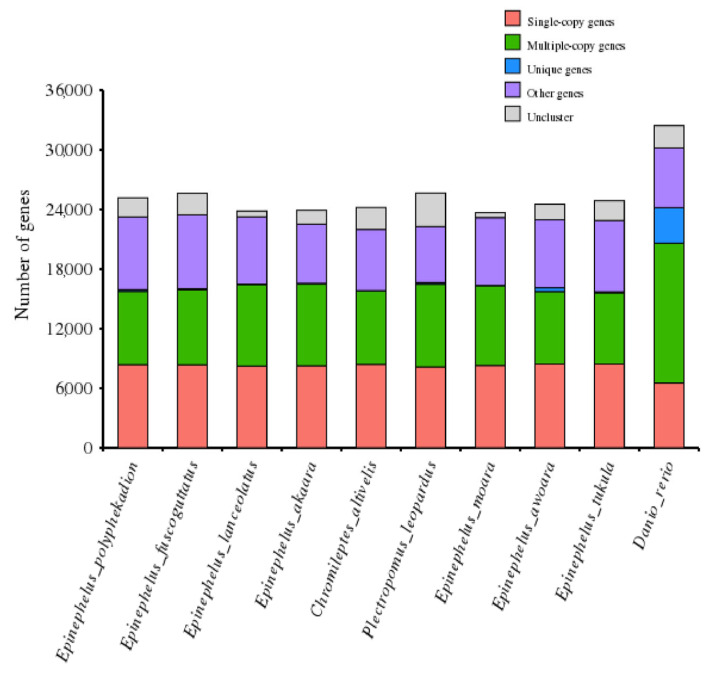
Statistical results for *Epinephelus* gene family.

**Figure A8:**
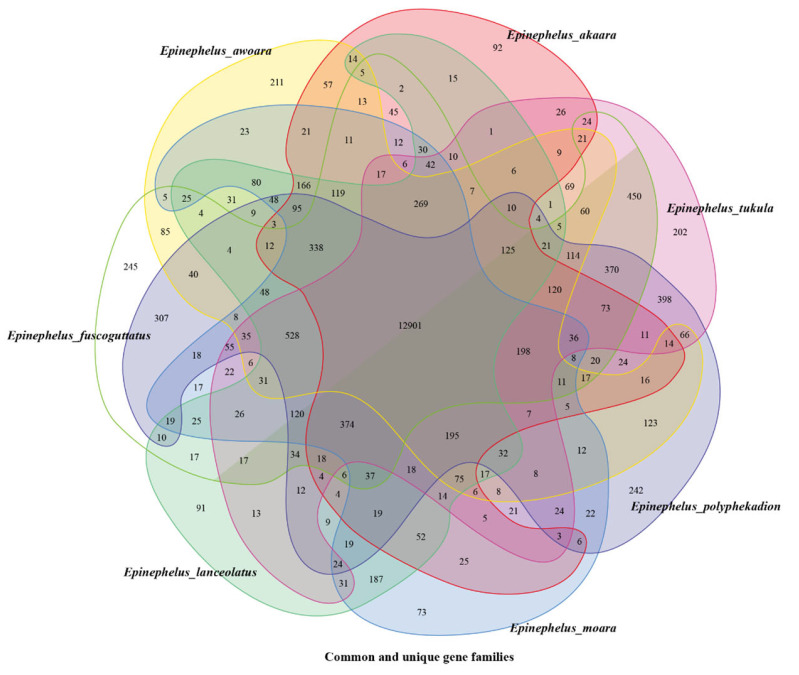
Venn diagram of *Epinephelus* gene family clustering.

Using *E. fuscoguttatus* and *E. polyphekadion* as target species, and *E. lanceolatus*, *E. akaara*, *E. moara*, *E. awoara*, *E. tukula*, *P. leopardus*, and *C. altivelis* as comparative species, along with the model organism *Danio rerio* as an outgroup, orthologs of the 10 fish species were predicted. A total of 253,963 genes were clustered into 21,710 gene families, of which 5457 were common single-copy gene families (Figure A7 and Figure A8). The shared single-copy genes were used to construct a phylogenetic tree with divergence times (Figure 3). Based on the 5457 shared single-copy gene families between the target species and comparative species, we analyzed the phylogenetic relationships and evolutionary history at the genomic level among the brown-marbled groupers, the camouflage groupers, and several other economically important groupers. The estimated divergence times indicate that the ancestor of groupers diverged from *Danio rerio* approximately 221.3 million years ago (204.3–252.1 million years). The divergence time among economically important groupers is more recent, with their ancestor beginning to diverge around 49 million years ago (40.8–59.0 million years) and leading to the formation of *P. leopardus* and other groupers. After the first divergence among groupers, approximately 23.5 million years ago (19.5–27.9 million years), the ancestors of the *E. awoara* and the *E. akaara* began to diverge, grouping them together. The genetic evolutionary relationship between the brown-marbled grouper and the camouflage grouper is the most recent among economically important groupers, with their ancestor diverging about 2.5 million years ago (2.0–3.2 million years).

### 2.5. Comparative Analyses of the Genome Structure

**Figure 4:**
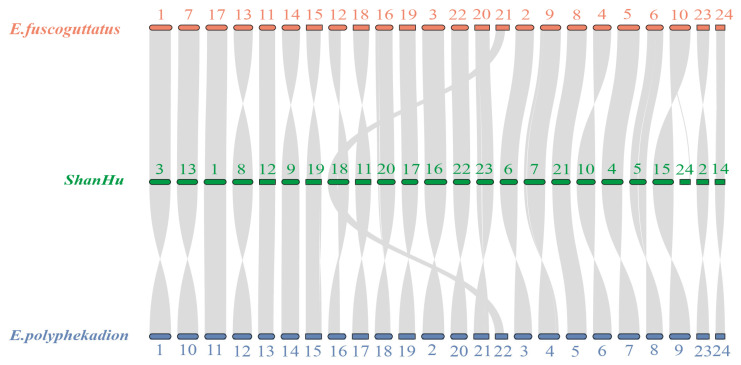
Collinearity comparison between the chromosomes of ShanHu, *E. fuscoguttatus*, and *E. polyphekadion*.

**Figure A9:**
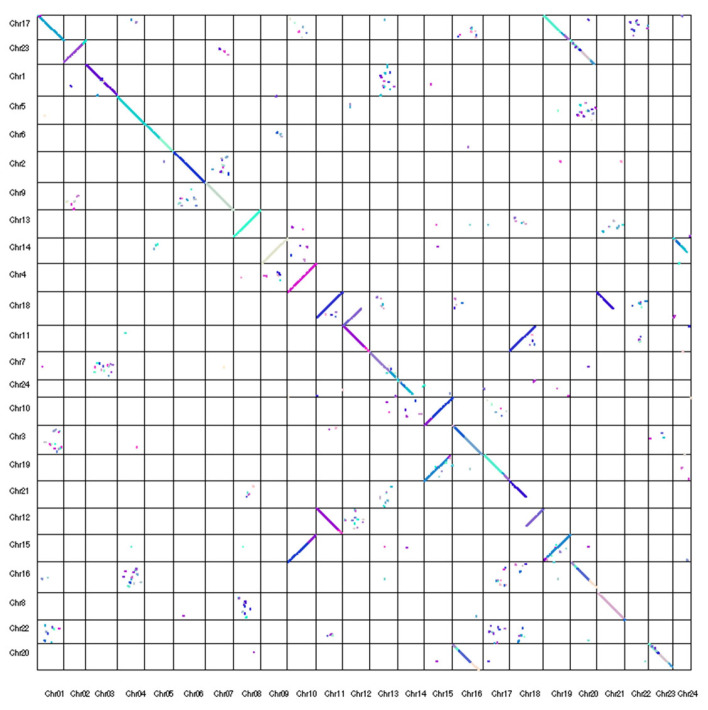
Whole genome alignment between ShanHu and *E. fuscoguttatus*. The vertical axis represents the *E. fuscoguttatus* and the horizontal axis represents the ShanHu.

**Figure A10:**
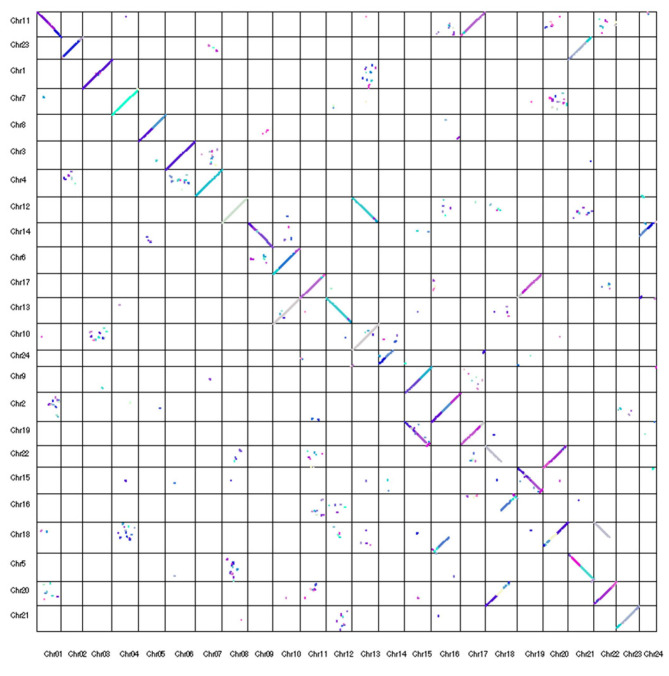
Whole genome alignment between ShanHu and *E. polyphekadion*. The vertical axis represents the E. polyphekadion and the horizontal axis represents the ShanHu.

A collinearity analysis was conducted on the ShanHu groupers genome in comparison with the brown-marbled groupers and camouflage groupers genomes to understand the structural differences in chromosomes between the hybrid ShanHu groupers and their maternal and paternal parents. (Figure 4). Between the genomes of hybrid ShanHu grouper and the maternal brown-marbled grouper, a total of 342 homologous gene collinear blocks were identified, with 73.87% of the genes located within these collinear blocks. While between the hybrid grouper and the paternal camouflage grouper genomes, 340 homologous gene collinear blocks were identified, with 76.43% of the genes falling within these blocks. The hybrid ShanHu grouper displayed a high proportion of homologous genes compared to both parental genomes (Figure A9 and Figure A10).

Both the ShanHu grouper vs. the maternal brown-marbled grouper and the ShanHu grouper vs. the paternal camouflage grouper exhibited vertical collinear regions, as well as collinear regions showing central inversion phenomena. The number of collinear genes between the ShanHu grouper and the camouflage grouper is higher than that between the ShanHu grouper and the brown-marbled grouper. Both the ShanHu grouper and the brown-marbled grouper, as well as the ShanHu grouper and the camouflage grouper, have vertical collinear regions and collinear regions with central inversion phenomena. There is a phenomenon of vertical collinearity in the structures of chromosomes No. 1, 3, 4, 5, 6, 7, 12, 13, 14, 16, 17, 20, 21, 22, and 23 of the ShanHu grouper and chromosomes No. 1, 2, 3, 5, 6, 7, 8, 9, 11, 16, 17, 19, 20, 22, and 24 of the brown-marbled grouper. Additionally, there is a phenomenon of vertical collinearity in the structures of chromosomes No. 1, 9, 12, 19, and 21 of the ShanHu grouper and chromosomes No. 5, 11, 13, 14, and 15 of the camouflage grouper. When comparing the hybrids, the structural similarity of the ShanHu groupers’ chromosomes to the maternal brown-marbled grouper was higher, while the proportion of homologous genes was greater for the ShanHu grouper compared to the paternal camouflage groupers despite exhibiting larger structural differences.

**Figure 5:**
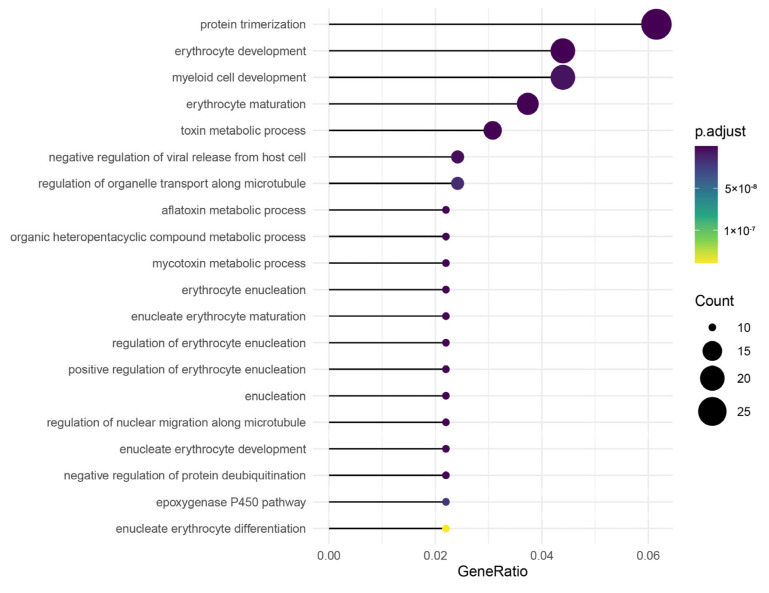
Enrichment of GO annotations with the expanded gene families in ShanHu (*p* < 0.05).

**Figure 6:**
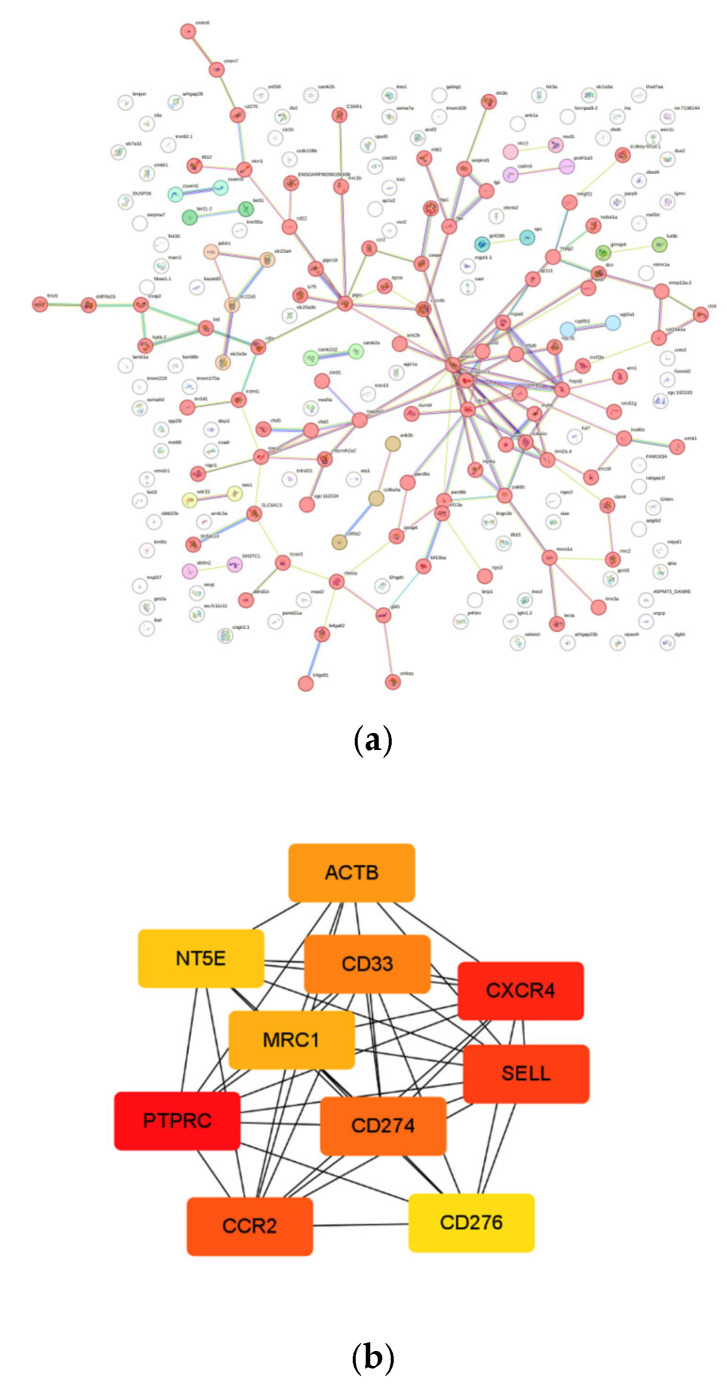
(**a**) Protein–protein interaction network analysis of expanded genes. (**b**) Identification of hub genes in terms of maximal clique centrality (MCC) score.

Comparative genomic analysis was conducted between the ShanHu grouper and the brown-marbled grouper and the camouflaged grouper. After hybridization, it was found that 1545 gene families had undergone expansion. GO functional enrichment was performed and multiple enriched pathways related to immunity and growth development were identified (Figure 5). The expanded genes were analyzed using protein–protein interaction (PPI) and maximal clique centrality (MCC) methods. Through the constructed PPI network it was found that several genes related to growth and immunity, such as *acta1b*, *hsp70.3*, *hspa8*, *cdc42*, and *cxcr4b*, are closely connected to other expanded genes. Furthermore, several hub genes, such as *cxc4*, *ptprc*, and *ccr2*, were identified as playing important roles among the expanded genes (Figure 6).

## 3. Discussion

The grouper is an important aquaculture fish species. Hybridization is an effective breeding method for improving the genetics and increasing production efficiency in fish farming practices, and it is also one of the most widely used breeding technologies in fish breeding. Currently, regarding grouper hybridization experiments, numerous research teams have conducted extensive studies and screened many promising grouper hybrid combinations, with the ShanHu grouper considered a hybrid grouper with significant development potential [18,21]. Whole genome sequencing represents the complete genetic material of an organism and is the most important means of understanding that organism’s information [22]. Deciphering the genome sequence of the ShanHu grouper can provide comprehensive reference data for studying the genetic mechanisms of grouper hybridization. Additionally, it can accelerate the foundational research on this species, which is substantially meaningful and practically valuable with respect to genetic trait analysis, genetic breeding, germplasm resource conservation, and aquaculture.

Firstly, based on the PacBio and Illumina sequencing platforms, as well as the PacBio SMRT technology and Hi-C assisted scaffolding technology, the whole-genome sequencing and assembly of the ShanHu grouper were carried out. The whole-genome size of the ShanHu grouper is 1.17 Gb, which is slightly larger than the genome size of its male parent, *E. fuscoguttatus*, at 1.08 Gb, and that of its female parent, *E. polyphekadion*, at 1.09 Gb. The scaffold N50 is 46.12 Mb and 92.04% of the sequences can be successfully anchored to the 24 chromosomes of the ShanHu grouper. On the basis of the completed genome assembly of the ShanHu grouper, we annotated its repetitive sequences, non-coding RNAs, and predicted 26,102 genes. Through the processes of sequencing, assembly, and annotation of the ShanHu grouper, complete whole-genome information of this hybrid grouper has been obtained, enabling a deeper understanding of the genetic basis of the ShanHu grouper which is a hybrid grouper with high economic value.

At the same time, a phylogenetic tree of economical groupers was constructed at the whole-genome level to understand the evolutionary relationships among groupers. It was found that the genetic evolutionary relationship between the brown-marbled grouper and the camouflage grouper is the closest among economical groupers, providing theoretical evidence for the high hybridization potential between them. Hybridization is recognized as a potentially creative force contributing to adaptation and species diversification [23]. The ShanHu grouper has a recombinant genome with the same ploidy level as its parental species. The ShanHu grouper is formed through homoploid hybrid speciation between the *E. fuscoguttatus* and the *E. polyphekadion*. It may take many forms with respect to its genomic makeup, ranging from the introgression of a single or few genes into a foreign genomic background to balanced genomic contributions from both parent lineages [24]. Synteny analysis between the ShanHu grouper and its maternal brown-marbled grouper and paternal camouflage grouper revealed a high proportion of homologous genes and structural variations, with centric inversions in the chromosomes of both the ShanHu grouper and the brown-marbled grouper as well as the camouflage grouper. There are 24,031 homologous genes between the ShanHu and the brown-marbled grouper, accounting for 92.0%, and 24,077 homologous genes between the ShanHu and the camouflage grouper, accounting for 92.2%. Between the genomes of the hybrid ShanHu grouper and the maternal brown-marbled grouper a total of 342 homologous gene collinear blocks were identified, with 73.87% of the genes located within these collinear blocks. While between the hybrid grouper and the paternal camouflage grouper genomes a total of 340 homologous gene collinear blocks were identified, with 76.43% of the genes falling within these blocks. More vertical synteny chromosomes were observed between the ShanHu grouper and the brown-marbled grouper compared to the camouflage grouper, whereas more centric inversion structural variations occurred between the ShanHu grouper and the camouflage grouper than between the ShanHu grouper and the brown-marbled grouper. The 8th, 9th, 10th, 11th, 15th, 18th, and 19th chromosomes of the ShanHu grouper showed structural central inversions with the 4th, 10th, 12th, 13th, 14th, 15th, and 18th chromosomes of the brown-marbled grouper. Additionally, the 3rd, 4th, 5th, 6th, 7th, 8th, 10th, 11th, 13th, 15th, 16th, 17th, 18th, 20th, 22nd, and 23rd chromosomes of the ShanHu grouper displayed central inversions with the 1st, 2nd, 3rd, 4th, 6th, 7th, 8th, 9th, 10th, 12th, 16th, 17th, 18th, 19th, 20th, and 21st chromosomes of the camouflage grouper. The chromosomal structure of the ShanHu grouper exhibits a greater degree of similarity to that of its maternal parent. Intriguingly, with respect to crucial aquaculture traits that are of primary concern for commercially valuable fish species, such as growth patterns, developmental characteristics, and morphological features, the ShanHu grouper also demonstrates a closer resemblance to its maternal parent. During the hybridization process of the ShanHu grouper’s parents, gene recombination occurs. The drastic changes in the genome will prompt the replication of some genes, thereby increasing the gene copy number. Based on the whole genome information, it was found that multiple genes related to immunity and growth development had expanded in the hybrid ShanHu grouper compared to in the parents. We speculate that during the hybridization process, gene recombination has led to the expansion of genes related to immunity and growth in the ShanHu grouper. This is an important reason why the ShanHu grouper demonstrates stronger resistance and growth advantages during the farming process.

The ShanHu grouper, as a new variety of groupers in the field of grouper aquaculture, has a very broad aquaculture prospect and is of far-reaching significance for our study of the genetic mechanism of grouper hybridization. Third-generation sequencing technology has been used to conduct whole-genome sequencing of the ShanHu grouper so as to obtain a whole-genome sequence of the hybrid grouper at the chromosomal level. Currently, many model organisms have completed whole genomes at the telomere-to-telomere level through the use of ultra-long sequencing technology, which enables more accurate and comprehensive coverage of the entire chromosomal region to obtain more complete assembled genome sequences and decipher complex genetic structures. With the improvement of sequencing technology and the popularization of ultra-long sequencing methods, in the future more complete whole genomes at the telomere-to-telomere level will emerge in the field of aquaculture to serve the development of the industry.

## 4. Materials and Methods

### 4.1. Sample Collection, Library Construction, and Sequencing

A ShanHu grouper was obtained by hybridizing the brown-marbled grouper as the maternal parent and the camouflage grouper as the paternal parent. The hybrid parents were sourced from Hainan Chenhai Aquatic Co., Ltd. (Lingshui), China. The hybrid groupers were raised to one year of age, and their heart, liver, spleen, kidney, skin, fins, gills, and muscle tissues were collected and immediately frozen in liquid nitrogen. Genomic DNA of the ShanHu grouper was extracted from dorsal muscle tissue using a QIAGEN DNeasy Kit (QIAGEN, Shanghai, China). For the Illumina platform (NEB, Ipswich, MA, USA), the short fragment library with an insertion size of 350 bp was generated using a NEB Next^®^ Ultra™ DNA Library Prep Kit. Subsequently, HiFi SMRTbell Libraries with an insert size of 20 kb were generated using the SMRTbell Template PrepKit 2.0 (PacBio, Menlo Park, CA, USA), and the library was sequenced in circular consensus sequencing (CCS) on the PacBio Sequel II platform. The Hi-C library was constructed following the standard protocol described previously with certain modifications and was sequenced using the Illumina NovaSeq 6000 platform [25]. In total, 59 Gb of Illumina data and 100 Gb of PacBio data were generated, and low-quality reads and sequencing-adaptor-contaminated reads were removed. RNA isolation was performed using RNAsimple Total RNA Kit (TIANGEN, Beijing, China). RNA-sequencing libraries for 8 tissues (spleen, gill, skin, muscle, liver, heart, kidney, and fin) were constructed using the NEBNext Ultra RNA Library Prep Kit following the manufacturer’s protocol and also sequenced on the Illumina NovaSeq 6000 platform.

### 4.2. Genome Size Estimation, Assembly, and Quality Assessment

First, the constructed library was sequenced using Illumina Novaseq for paired-end (PE) sequencing. To ensure the quality of the data analysis, raw reads were filtered to obtain clean reads. *K*-mer frequency distribution analysis was then used to estimate the genome characteristics using Jellyfish (v2.2.7), based on a 17-mer [26]. The estimated genome size was 1125.98 Mb, the heterozygosity rate was approximately 1.7%, and the repeat content was 41.12%. The genome was then assembled using SOAPdenovo2 (r242) software with a 41-mer to obtain the preliminary assembly results for the ShanHu grouper genome [27]. Based on the pre-assembly results, sequencing was performed on the PacBio platform with a coverage depth of 88.89×. Quality control was conducted on the raw sequencing data to obtain HiFi reads, and the genome was assembled using Hifiasm (v0.19.5) software [28]. Finally, the completeness of the genome assembly was assessed using Benchmarking Universal Single-Copy Orthologs (BUSCO) (v4.1.2) [29].

### 4.3. Pseudochromosome Construction

Following the standard protocol described previously with certain modifications, we constructed Hi-C libraries using the original sample as input [30]. Hi-C sequencing was performed using muscle tissue from the ShanHu grouper. Based on the principle that inter-chromosomal interactions are weaker than intra-chromosomal interactions and that long-range interactions are weaker than short-range ones, we used high-throughput chromosome conformation capture (Hi-C) technology to cluster contigs into chromosome groups. The order and orientation of the contigs within each chromosome group were then determined to construct the chromosome-level whole genome of the ShanHu grouper. Based on the sequencing results and the obtained Hi-C reads, the contig sequences assembled were mapped to the chromosome level using Allhic (v0.9.8) software [31].

### 4.4. Genome Prediction and Annotation

We conducted repeat annotation, non-coding RNA annotation, protein-coding gene prediction, and functional annotation on the ShanHu grouper genome. For repeat annotation, repetitive sequences were detected using a combined approach of homology alignment and de novo searches. Homologous sequences comparison was conducted using RepeatMasker (v4.1.0) and RepeatProteinMask based on Repbase database [32]. And a de novo candidate database of repetitive elements was constructed by LTR (v1.0.6), RepeatScout (v1.0.5), and RepeatModeler (v2.0.1) software [33,34,35]. Additionally, tandem repeat sequences were predicted using the software TRF (v4.09). Non-coding RNA annotations, including rRNA, snRNA, and microRNA, were identified using Infernal (v1.1) and tRNAs were predicted using tRNAscan- SE (v2.0) [36,37]. A combination of homology-based prediction, de novo prediction, and transcriptome-based prediction was used to predict the protein-coding genes within the ShanHu grouper genome. First, protein sequences from other fish, including *E. fuscoguttatus*, *E. lanceolatus*, *E. moara*, *Danio rerio*, *Gasterosteus aculeatus*, and *Takifugu rubripes*, were aligned against the ShanHu grouper genome using Augustus (v3.2.3), Glimmer (v3.0.4), and Genscan (v1.0) software [38,39,40]. Next, solar software (v0.9.6) was used to conjoin with BLAST (v2.2.26) to generate a hit. GeneWise (v2.4.1) was then used to predict the exact gene structure of the corresponding genomic region on each BLAST hit [41,42]. Additionally, high-quality RNA-seq data were assembled by Trinity (v2.11.0), and the sequences were aligned against the ShanHu grouper genome to assemble spliced alignment [43,44]. Furthermore, RNA-seq reads were mapped to the ShanHu grouper genome using Tophat (v2.0.13) and then assembled into gene models by Cufflinks (v2.1.1) [45]. Finally, a non-redundant and more complete gene set was integrated via EvidenceModeler (EVM) (v1.1.1) using various gene models. Furthermore, the predicted protein-coding genes in the ShanHu grouper genome were annotated based on public databases including SwissProt, NR database, InterPro, and KEGG pathway [46,47,48].

### 4.5. Phylogenetic Analysis

Using the brown-marbled grouper and the camouflage grouper as target species, a phylogenetic tree for groupers was constructed to understand the evolutionary relationships between the brown-marbled grouper and the camouflage grouper in relation to other economically important grouper species. First, orthologous relationships between the genes of *E. lanceolatus*, *E. akaara*, *E. moara*, *E. awoara*, *E. tukula*, *P. leopardus*, *C. altivelis*, and *D. rerio* were inferred through all-against-all protein sequence similarity searches with OthoMCL (v1.4), retaining only the longest predicted transcript per locus, with an E-value threshold set to 1 × 10^−5^ [49,50]. Then, for each gene family, an alignment was produced using Muscle (v3.8.31), ambiguously aligned positions were trimmed using Gblocks (v0.91b), and the tree was inferred using RAxML (v8.2.12) [51,52]. Divergence times between species were calculated using the MCMCtree (v1.1) program implemented in PAML [53].

### 4.6. Comparative Genomic Analyses

Based on the whole-genome information of the brown-marbled grouper, the camouflage grouper, and the hybrid ShanHu grouper, synteny analysis was conducted using MCscanX software (E-value ≤ 1 × 10^−5^, match size ≥ 5) with default parameters [54]. We identified the orthologous groups among these three species using all-to-all blast (E-value ≤ 1 × 10^−5^, identity ≥ 80%) and identified the expanded families using café [55]. Enrichment analyses based on GO annotations were performed to identify functional implications of the expanded family (Fisher’s exact test, adjusted *p*-value < 0.05). A PPI network was conducted using STRING v11.0 with default parameters [56]. Additionally, the maximal clique centrality (MCC) method of the cytoHubba module in Cytoscape (v3.10.3) software was performed to determine the hub genes in the networks [57].

## Figures and Tables

**Table 1:** Genome assembly statistics for the hybrid ShanHu grouper.

Type	*E fuscoguttatus*	*E. polyphekadion*	ShanHu
Total length of Contigs (bases)	1,082,718,103	1,099,728,802	1,176,882,344
Number of Contigs	396	164	171
Max length Contig (bases)	52,327,456	53,203,867	58,196,232
Contig N50 (bases)	37,367,964	40,804,240	45,365,500
Contig N90 (bases)	9,232,285	12,950,428	15,571,449
Total length of Scaffolds (bases)	1,082,726,703	1,099,733,602	1,176,892,644
Number of Scaffolds	310	116	68
Scaffold N50 (bases)	44,785,048	47,393,752	46,120,958
Scaffold N90 (bases)	39,728,480	39,964,760	24,826,122

**Table 2:** Statistics of gene predictions in the hybrid ShanHu genome.

Type	Gene Set	Number	Average Transcript Length (bp)	Average CDS Length (bp)	Average Exons Per Gene	Average Exon Length (bp)	Average Intron Length (bp)
De novo	Augustus	39,878	10,427.62	1197.88	6.60	181.45	1647.61
	SNAP	41,336	39,332.49	1185.35	8.12	145.91	5355.06
Homolog	*E.fuscoguttatus*	25,123	17,952.51	1712.51	9.69	176.79	1869.54
	*E.lanceolatus*	24,336	17,919.25	1698.19	9.68	175.49	1869.42
	*E.moara*	24,927	17,676.76	1694.95	9.63	176.05	1852.45
	*T. rubripes*	21,740	17,560.21	1688.49	9.49	177.94	1869.64
	*G. aculeatus*	22,296	17,691.72	1702.10	9.56	178.12	1868.87
	*D.rerio*	22,09	15,746.66	1541.84	8.50	181.39	1893.95
RNA-seq	Transcripts	106,29	4100.74	898.00	2.50	151.12	1749.27
	PASA	21,723	7242.56	715.03	4.73	177.73	1876.96
EVM	EVM	35,887	13,394.27	1320.96	7.43	177.73	1876.96
Pasaupdate	pasaupdate	35,739	13,629.09	1326.57	7.47	177.64	1902.11
Final set	Final	26,102	17,514.95	1626.80	9.43	172.60	1885.81

**Table 3:** Annotation results of repetitive sequences in ShanHu genome.

Type	Number	Length (bp)	Percentage (%)
SINEs	11,410	905,388	0.08
LINEs	328,957	91,778,626	7.80
LTRs	563,312	103,517,634	8.80
DNA	1,733,660	316,822,578	26.92
Unknown	106,739	16,787,622	1.43
Total		552,112,951	46.91

**Table 4:** Annotation results for non-coding RNA in ShanHu genome.

Type		Copy (w)	Total Length (bp)	% in Genome
miRNA		3438	471,917	0.040%
tRNA		2415	180,826	0.015%
rRNA	rRNA	4187	1,069,967	0.091%
	18s	445	279,049	0.024%
	28s	1058	474,992	0.040%
	5.8s	145	22,458	0.002%
	5s	2539	293,468	0.025%
snRNA	SnRNA	836	121,144	0.010%
	CD-box	171	19,670	0.002%
	HACA-box	95	14,842	0.001%
	splicing	504	74,708	0.006%
	scaRNA	65	11,867	0.001%

## Data Availability

Whole Genome Shotgun project for ShanHu has been deposited at GenBank under the accession GCA_047716675.1 under BioProject number PRJNA1210092.

## Appendix A

**Table A1:** Scaffold Cluster Number and Length of Each Chromosome in ShanHu.

Sequeues ID	Cluster Number	Sequeues Length (bp)
Chr1	1	44,551,120
Chr2	2	37,351,789
Chr3	1	53,015,742
Chr4	1	47,211,449
Chr5	1	48,719,712
Chr6	1	53,326,279
Chr7	1	48,243,466
Chr8	1	46,120,958
Chr9	1	45,365,500
Chr10	1	48,494,318
Chr11	1	43,829,164
Chr12	1	43,297,142
Chr13	1	47,782,323
Chr14	1	24,826,122
Chr15	1	48,046,878
Chr16	1	52,374,887
Chr17	1	41,935,179
Chr18	1	58,196,232
Chr19	1	45,048,601
Chr20	1	45,100,459
Chr21	2	46,795,679
Chr22	2	39,857,385
Chr23	25	41,020,352
Chr24	100	32,676,861
